# Water use strategies and coexistence mechanisms of desert plants in relation to groundwater depth: evidence from stable oxygen isotope

**DOI:** 10.3389/fpls.2026.1896220

**Published:** 2026-09-01

**Authors:** Zhao Jie Chen, Xue Wu, Lu Gong, Jia Nan Zhang, Chang Liang Shao

**Affiliations:** 1 College of Ecology and Environment, Xinjiang University, Urumqi, Xinjiang, China; 2 Key Laboratory of Oasis Ecology, Ministry of Education (Xinjiang University), Urumqi, Xinjiang, China; 3 Geological Bureau, Xinjiang Uyghur Autonomous Region, Urumqi, Xinjiang, China; 4 Management Center of Xinjiang Kalamaili Mountain Ungulate Nature Reserve, Changji, Xinjiang, China

**Keywords:** groundwater depth, hydrological niche differentiation, Kalamaili National Park candidate area, stable isotope, water use

## Abstract

Understanding the mechanisms of plant coexistence and vegetation -groundwater interactions in arid regions is critical for predicting ecosystem responses to environmental change. Groundwater depth is a key factor affecting plant water availability, yet how changes in groundwater accessibility shape the water-use strategies and niche differentiation of coexisting desert species remains unclear. We investigated three typical desert species (*Haloxylon persicum, Calligonum leucocladum*, and *Haloxylon ammodendron*) in the Kalamaili National Park candidate area, Xinjiang, China. Using stable oxygen isotope (δ^18^O) techniques combined with a mixing model, we analyzed plant water sources and use strategies across three phenological phases (May: leaf-out; July: peak physiological stress; September: pre-senescence), and examined the effects of groundwater depth on water-use patterns. Soil water isotope composition showed pronounced seasonal variation, with depletion in shallow layers (0 -40 cm) in spring and enrichment in summer and early autumn, while deep layers (100 -300 cm) remained relatively stable. The three species exhibited markedly different water-use strategies: *H. ammodendron* relied predominantly on groundwater (mean contribution: 37% -73% across seasons); *C. leucocladum* relied mainly on shallow and middle soil water (29% in spring, 31% in summer), consistent with its shallow but laterally extensive root system; *H. persicum* used both deep soil water and groundwater, but with lower groundwater dependence. Groundwater use proportion of *H. ammodendron* showed a significant negative correlation with groundwater depth (*P* < 0.05), while those of the other two species were largely decoupled from groundwater. Our findings suggest that hydrological niche segregation among coexisting desert species may arise from asymmetric groundwater sensitivity --only the phreatophyte closely tracks groundwater fluctuations, whereas others compensate through flexible use of seasonally variable soil water --thereby reducing competition and stabilizing community coexistence. We propose this asymmetric response as a mechanism for transient coexistence or short-term niche differentiation in groundwater-dependent ecosystems, providing a conceptual framework for vegetation restoration and water resource management.

## 1 Introduction

Arid regions, covering more than 40% of the global land area, are characterized by scarce precipitation and strong evaporation, making water availability a critical factor limiting plant survival, growth, and distribution (Maestre et al., 2025; Sardans et al., 2024). Plants, as primary producers, determine ecosystem responses to water availability through their water use patterns (Hu et al., 2024; Morgan et al., 2025). In arid ecosystems, plants absorb soil water or groundwater to maintain photosynthesis, growth, and development (Ruehr et al., 2023; Wang et al., 2023). Changes in water availability directly affect transpiration, stomatal conductance, and carbon assimilation, thereby regulating ecosystem carbon and water cycles (Fatichi et al., 2016; Liang et al., 2024; Zhang et al., 2026). In desert ecosystems without groundwater recharge, plants rely mainly on precipitation-derived soil water, where water stress significantly restricts vegetation growth by limiting transpiration and carbon assimilation. Conversely, in areas with groundwater recharge, groundwater mitigates drought impacts by sustaining soil moisture in the root zone, thereby maintaining transpiration and carbon assimilation, and ultimately promotes plant growth (Nan et al., 2025). Thus, understanding plant water use mechanisms is essential for revealing ecosystem stability and guiding ecological restoration in arid regions (Wang et al., 2026).

Accurately quantifying plant water sources is essential for understanding plant water use strategies (Muñoz-Gálvez et al., 2025). Traditional methods, such as root excavation, are destructive, time-consuming, and fail to capture the dynamics of actual water uptake (de la Casa et al., 2021; Lei et al., 2022). The advent of stable hydrogen and oxygen isotope (*δ*²H and *δ*^18^O) tracing techniques has revolutionized this field. Because different water sources (precipitation, soil water, groundwater) have distinct isotopic compositions, and plant roots typically do not cause isotopic fractionation during water uptake (except in certain groups such as halophytes), the proportional contributions of different sources can be quantitatively determined by comparing the isotopic composition of xylem water with that of potential sources (Dawson et al., 2002; Eheringer and Dawson, 1992; Xu et al., 2025). Owing to their high sensitivity and tracing capability, hydrogen and oxygen isotope techniques have become important tools for studying water transport along the soil-plant-atmosphere continuum (Cao et al., 2025; Liu et al., 2023). Recent studies have applied mixing models (e.g., MixSIAR, IsoSource) to distinguish plant water sources across multiple temporal scales and ecological levels (Barbeta et al., 2022). These isotopic techniques have therefore deepened our understanding of plant–water relations, revealing the functional plasticity of root water uptake and the hydrological niches of coexisting species under varying environmental conditions (Fan et al., 2025).

How multiple plant species coexist in resource-limited environments is a central question in ecology (Broekman et al., 2019; Silvertown, 2004). According to the competitive exclusion principle, species competing for the same limited resources cannot persist together over the long term (Hardin, 1960). However, in arid ecosystems, the coexistence of multiple plant species within the same area is commonly observed (Cai et al., 2025; Pérez-Ramos et al., 2019). A large body of research has shown that niche differentiation based on water use is critical for mitigating interspecific competition and facilitating species coexistence, and hydrological niche segregation (HNS) is considered a key mechanism underlying this process (Song et al., 2022). Hydrological niche differentiation encompasses three aspects: (1) partitioning of the soil moisture gradient at fine spatial scales; (2) partitioning of water resources through different water acquisition strategies; and (3) staggered regeneration opportunities created by temporal storage effects (Silvertown et al., 2015). Extensive field evidence indicates that coexisting plants can reduce inter-specific competition through differential root distribution, phenological characteristics, and water use strategies. Pronounced spatiotemporal differences in water uptake have been observed among coexisting plants in the semi-arid grasslands of Nebraska, the tropical dry forests of Panama, the subtropical karst forests of China, and the Badain Jaran Desert of Northwest China (Eggemeyer et al., 2009; Nie et al., 2011; Fan et al., 2025). As a representative example, at the southern margin of the hyper-arid Badain Jaran Desert, coexisting shrubs *Reaumuria songarica* and *Nitraria sphaerocarpa* exhibit distinct and flexible water use strategies, as they both responded only to larger precipitation pulses, but *N.sphaerocarpa* exhibited higher uptake intensity and longer reliance duration on shallow water sources post-precipitation (Gu et al., 2025; Fan et al., 2025). Similarly, in karst rocky desertification areas, trees and shrubs achieve coexistence by utilizing fissure water at different depths, with trees relying more on deep fissure water and groundwater (Cai et al., 2025). Vertical spatial hydrological niche differentiation could effectively avoids direct competition for limited water among species. Temporal separation is equally important, for instance, winter annuals adjust their germination ratios between autumn and spring to achieve intraspecific niche differentiation over their life cycle, thereby buffering inter-annual competitive pressure (Chesson et al., 2004). Furthermore, hydrological niche separation is not static but shifts dynamically with water availability (Fan et al., 2025). These studies reveal that hydrological niche differentiation is an important mechanism for maintaining plant species diversity and community stability in arid regions (Zhou et al., 2026).

In arid regions, groundwater serves as a critical water source for maintaining ecosystem function. Changes in groundwater depth exert significant influences on plant water use (Liu et al., 2017). When the water table is shallow, groundwater can replenish soil moisture through capillary rise, providing a stable water supply for plants (Zeng et al., 2020). As groundwater table declined, this recharge effect weakens, forcing plants either to extend their root systems to deeper layers at greater energy cost or to shift their reliance to precipitation-derived shallow soil water (Rong et al., 2025). Different plant species exhibit species-specific responses to variations in groundwater depth, reflecting differences in root plasticity and hydrological niche breadth (Shi et al., 2022). These differential responses provide important insights for vegetation restoration and water resource management in arid regions: appropriate plant configurations should be selected according to local groundwater conditions to improve water use efficiency and ensure the sustainability of ecological restoration.

Kalamaili National Park candidate area is situated in the transitional zone between the Junggar Basin and the northern piedmont of the Tianshan Mountains, possessing distinctive regional characteristics and significant ecological conservation value (Li et al., 2025a). This region features a typical temperate hyper-arid desert climate, characterized by scarce precipitation, intense evaporation, and extreme scarcity of surface water resources, rendering groundwater the most critical water source sustaining regional vegetation survival and stability. The terrain within the area is complex, with intertwined distributions of low‐mountain hills, denudation plains, and sandy deserts, fostering a variety of xerophytic and hyper-xerophytic shrub communities (Li et al., 2025b). These plants not only constitute the main body of the regional ecological barrier but also provide critical habitats for rare and endangered ungulates, including the *Equus hemionus* and the *Gazella subgutturosa* (Li et al., 2025a; Shao et al., 2025). In recent years, intensified climate change and increased anthropogenic activities, such as mining and road construction, have led to continuous fluctuations in groundwater depth, posing potential threats to the health of desert vegetation (Shao et al., 2025). Signs of vegetation degradation have emerged in some areas; however, the underlying ecohydrological mechanisms remain poorly understood—specifically, how different species temporally adjust their water sources in response to seasonal drought, whether coexisting species maintain stable hydrological niche differentiation to alleviate competition, and how groundwater table dynamics influence these critical water-use strategies. These questions currently lack systematic evidence based on stable isotope hydrology.

Based on the research background outlined above, this study takes the Kalamaili National Park candidate area as the study area. Using stable hydrogen and oxygen isotope techniques, we investigate the water sources and seasonal dynamics of different desert plant species, reveal the mechanisms of hydrological niche differentiation among coexisting plants, and elucidate the effects of groundwater depth on plant water use, with the aim of providing a scientific basis for ecological conservation and vegetation restoration in arid regions.

## 2 Materials and methods

### 2.1 Study sites and plant species

Kalamaili National Park candidate area (88°26′–90°09′ E, 44°38′–46°03′ N) is located in the interior of the Eurasian continent, on the eastern margin of the Junggar Basin in Xinjiang, covering an area of approximately 1.47 * 10^4^ km² (Shao et al., 2025). The terrain exhibits a vertical zonation from the southeast and northeast toward the west, with elevations ranging from 430 m to 1,464 m. The southern part consists of the Gurbantunggut Desert and the piedmont Gobi of the Kalamaili Mountains, the central part is the Kalamaili mountain area, and the northern part is a low mountain and hilly region (Guan et al., 2025). The climate is typical of a temperate continental arid climate, with cold winters (average January temperature below -20°C) and hot summers (average July temperature between 33°C). Over the past 20 years, the local annual average temperature has been 6.36°C, and the annual average precipitation has been 166.39 mm (Li et al., 2025a). The vegetation is primarily composed of shrub communities dominated by *Haloxylon* spp. and *Calligonum* spp., semi-shrubs such as *Ceratoides latens* and *Anabasis* spp., desert herbaceous plants including *Stipa capillata* and *Stipa glareosa*, and halophytes represented by *Kalidium foliatum* and *Suaeda* spp (Li et al., 2025b). The vegetation is relatively sparse, with a simple community structure.

**Figure 1:**
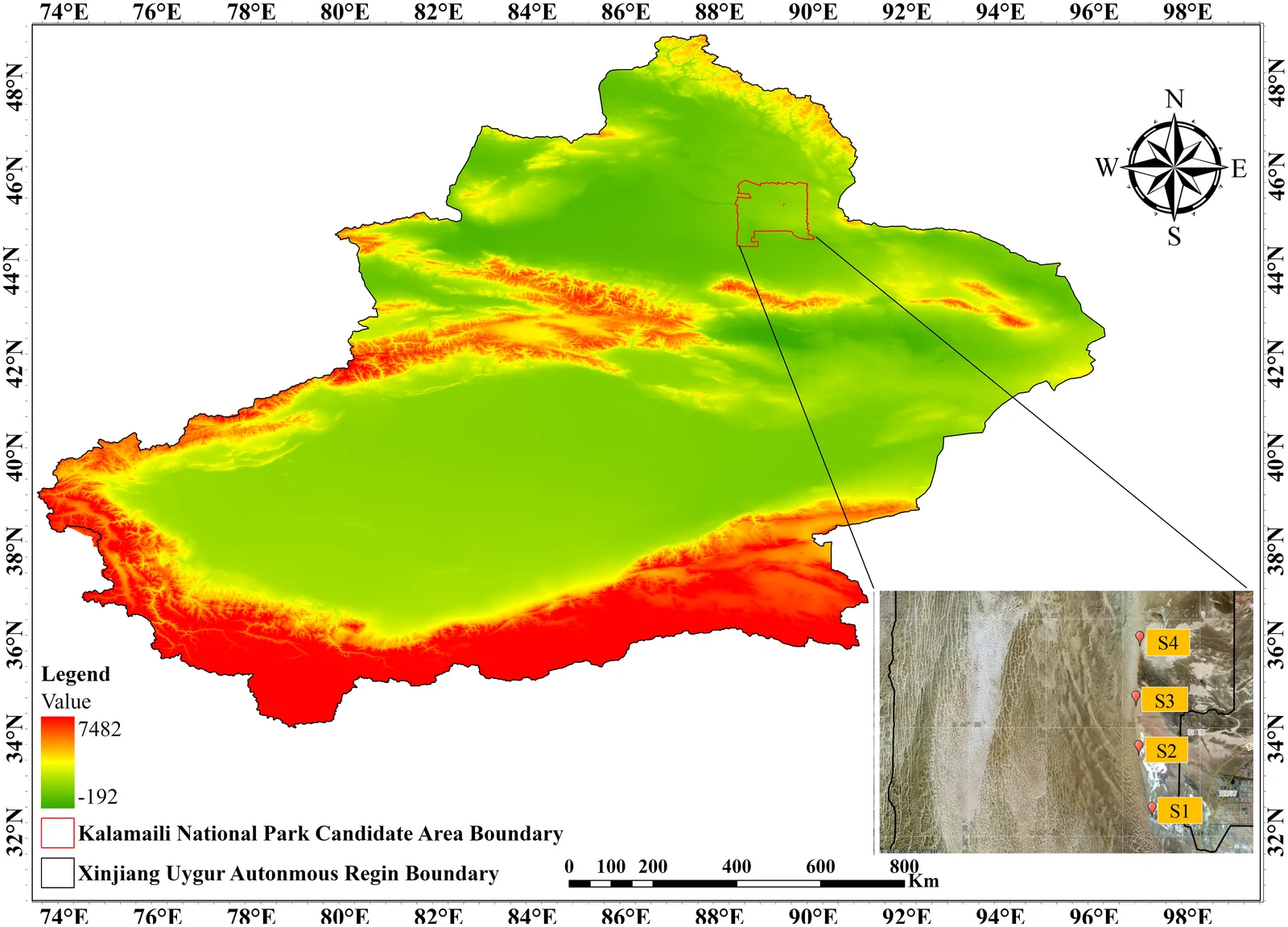
Location of the study area.

The study area lies in a historically mining-focused region where available hydrogeological data primarily address deep confined groundwater (exceeding 100 m) rather than shallow phreatic water accessible to plants. Natural topographic variability creates a groundwater depth gradient from the edge to the interior of the Kalamaili National Park candidate area. Along this gradient, we studied four inter-dune lowland sites with groundwater depths of 1.5, 3, 4.5, and 6.4 m (namely S1: GW = 1.5m, S2: GW = 3.0m, S3: GW = 4.5m, S4: GW = 6.4m) (**Figure 1**). These gradients were selected based on observable differences in vegetation growth and community structure across depths, and precipitation input was comparable among the sites within each season, making them especially well-suited for investigating plant water-use patterns in relation to groundwater depth. Vegetation distribution and groundwater chemical properties across the sampling plots are presented in **Tables 1**, **2**, respectively.

**Table 1:** Vegetation distribution characteristics at different groundwater depths.

Index	GW=1.5m	GW=3.0m	GW=4.5m	GW=6.4m
Coverage	57%	32%	53%	40%
Margalef richness	0.49	0.64	0.58	0.34
Shannon diversity	0.51	0.64	0.58	0.34
Pielou evenness	0.63	0.59	0.43	0.27
Simpson diversity	0.73	1.25	0.90	0.68

**Table 2:** Chemical properties of groundwater at different groundwater depths.

Index	Unit	GW=1.5m	GW=3.0m	GW=4.5m	GW=6.4m
pH	–	7.42	8.11	7.40	8.32
Turbidity	NTU	0.90	23.00	100	0.90
K*^+^*	mg/L	6.20	2.20	67.20	8.80
Na^+^	mg/L	3875.40	488.50	4777.74	3504.90
Ca^2+^	mg/L	120.20	120.20	2805.60	138.30
Mg^2+^	mg/L	109.40	24.30	266.33	77.80
Cl^-^	mg/L	5315.80	478.60	8862.50	5388.40
SO_4_^2-^	mg/L	1873.20	696.40	5004.70	629.20
CO_3_^2-^	mg/L	12.00	7.00	0.00	6.00
HCO_3_^-^	mg/L	152.60	79.30	61.00	207.50

### 2.2 Analysis of the *δ*^18^O of xylem water and water sources

The dominant species (*H. persicum*, *C. leucocladum*, and *H. ammodendron*) were selected as the research subjects (**Table 3**). One typical 10 m × 10 m plot was established at each of four groundwater depths along the gradient. In each plot, three healthy, similarly-sized individuals per species were marked for repeated sampling. For each species at each plot, xylem samples were collected from all three marked individuals (n = 3); soil samples were collected with three replicates per depth interval per plot (n = 3); and groundwater samples were collected in triplicate from each observation well (n = 3). Plant, soil, and groundwater samples were collected in May, July, and September 2025, with each sampling campaign conducted after multiple consecutive rainless days (≥7 days without measurable precipitation (>0.5 mm)) to minimize the potential influence of recent precipitation on isotopic signatures. For each marked plant, suberized twigs (3–4 cm long) were sampled from four directions (east, south, west, and north) between 08:00 and 09:00 h local time using clippers. Xylem samples were immediately sealed in vials, wrapped with Parafilm. Soil samples were collected beneath the canopy of each target plant using a hand auger. At the three deeper sites (groundwater depth ≥ 3 m), samples were taken from ten depth intervals: 0–20, 20–40, 40–60, 60–80, 80–100, 100–140, 140–180, 180–220, 220–260, and 260–300 cm. At the shallowest site (1.5 m depth), sampling was limited to seven intervals down to 150 cm (0–20, 20–40, 40–60, 60–80, 80–100, 100–140, and 140–150 cm). Each soil sample was split into two portions: one portion was sealed in a bag for isotope analysis; the other was placed in a tin box to determine gravimetric soil water content (SWC, %) by oven-drying at 105 °C for 24 h. Groundwater samples were collected from nearby observation wells. All samples (soil, xylem, and groundwater) were immediately placed in a portable ice cooler (Nylex, ESKY) to prevent evaporation, transported to the laboratory upon completion of field sampling, and stored at below –20 °C until subsequent isotope extraction, whereas groundwater samples were refrigerated at 4 °C and analyzed as soon as possible to minimize any chemical or isotopic alteration.

**Table 3:** Basic information of the study species.

Feature	*H. persicum*	*C. leucocladum*	*H. ammodendron*
Family	Amaranthaceae	Polygonaceae	Amaranthaceae
Life form	Shrub or small tree	Perennial shrub	Shrub or small tree
Habitat preference	Mobile and semi-mobile sand dunes	Fixed sand dunes, active sand dunes	Interdune lowlands, fixed sand dunes
Rooting pattern	Deep taproot reaching groundwater; lateral roots concentrated in 0–1.5 m	Lateral roots mainly in shallow layer (10–20 cm), extending >20 m horizontally	Deep taproot with horizontal roots extending >10 m
Root system type	Dual-root system (uses both shallow soil water and deep groundwater)	Shallow-spreading root system with extensive lateral spread	Deep-spreading root system
Leaf characteristics	Assimilating branches perform photosynthesis; leaves reduced	Leaves highly reduced; assimilating branches with thick cuticle	Leaves reduced to tiny scales
Picture	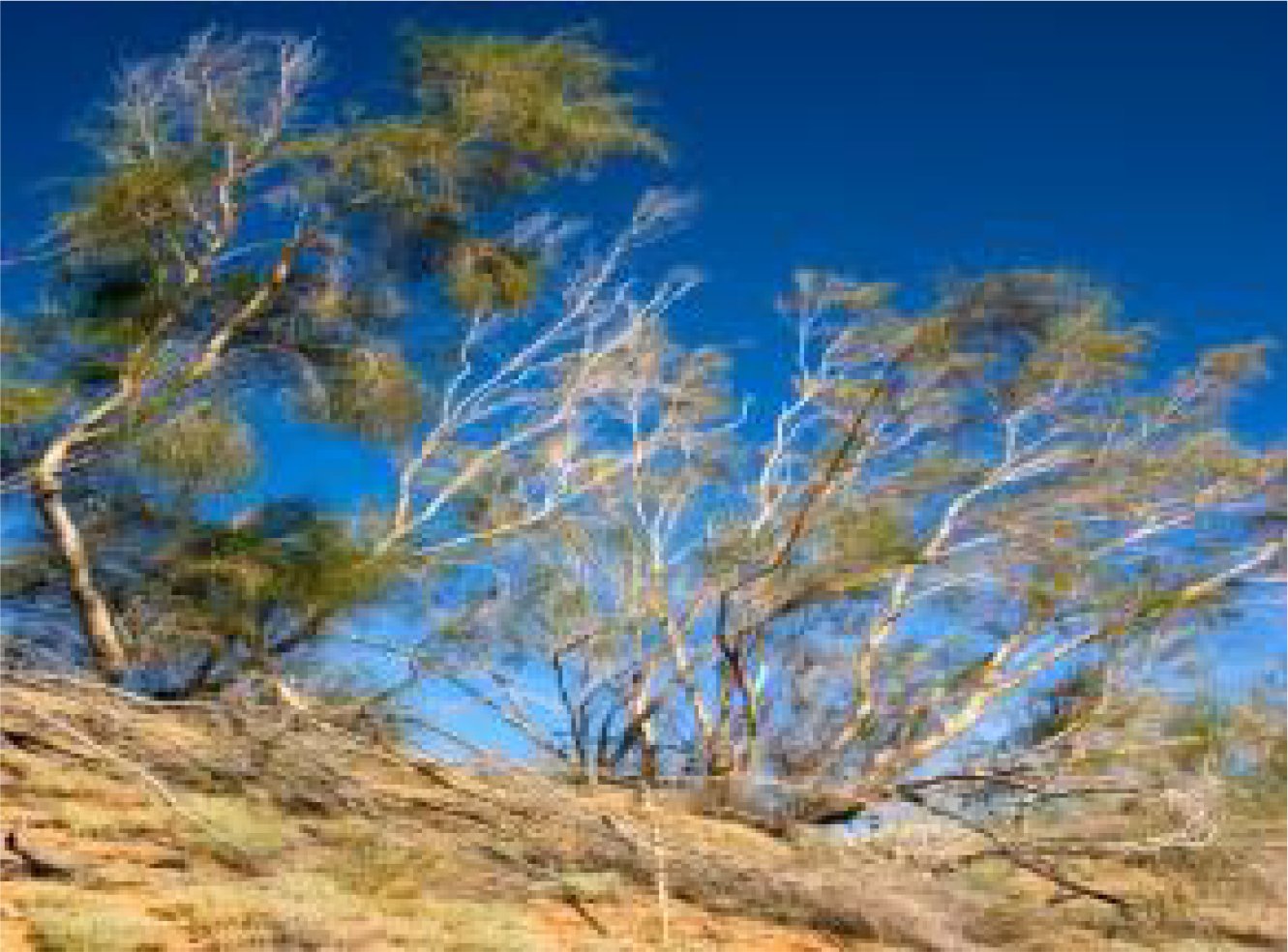	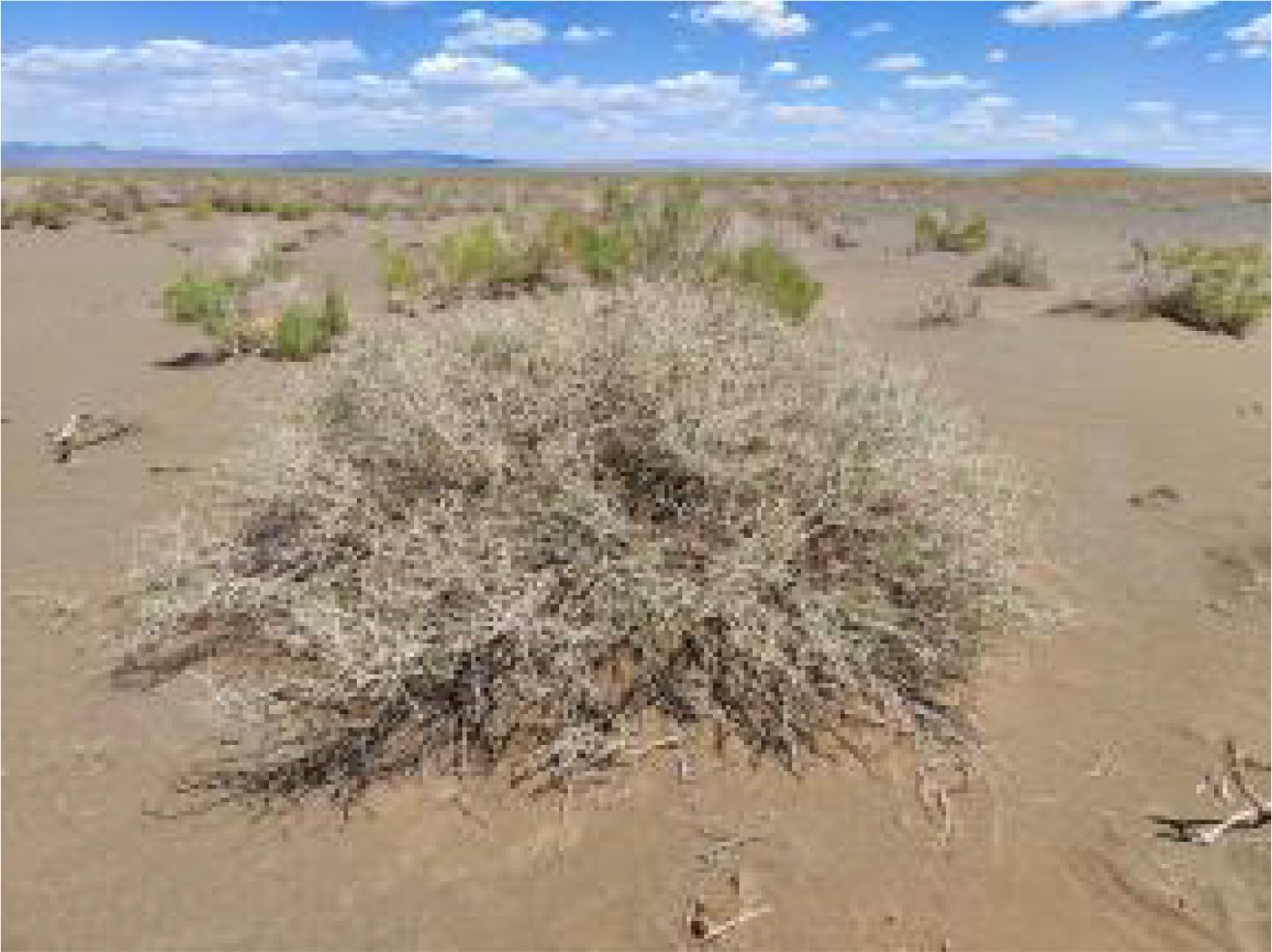	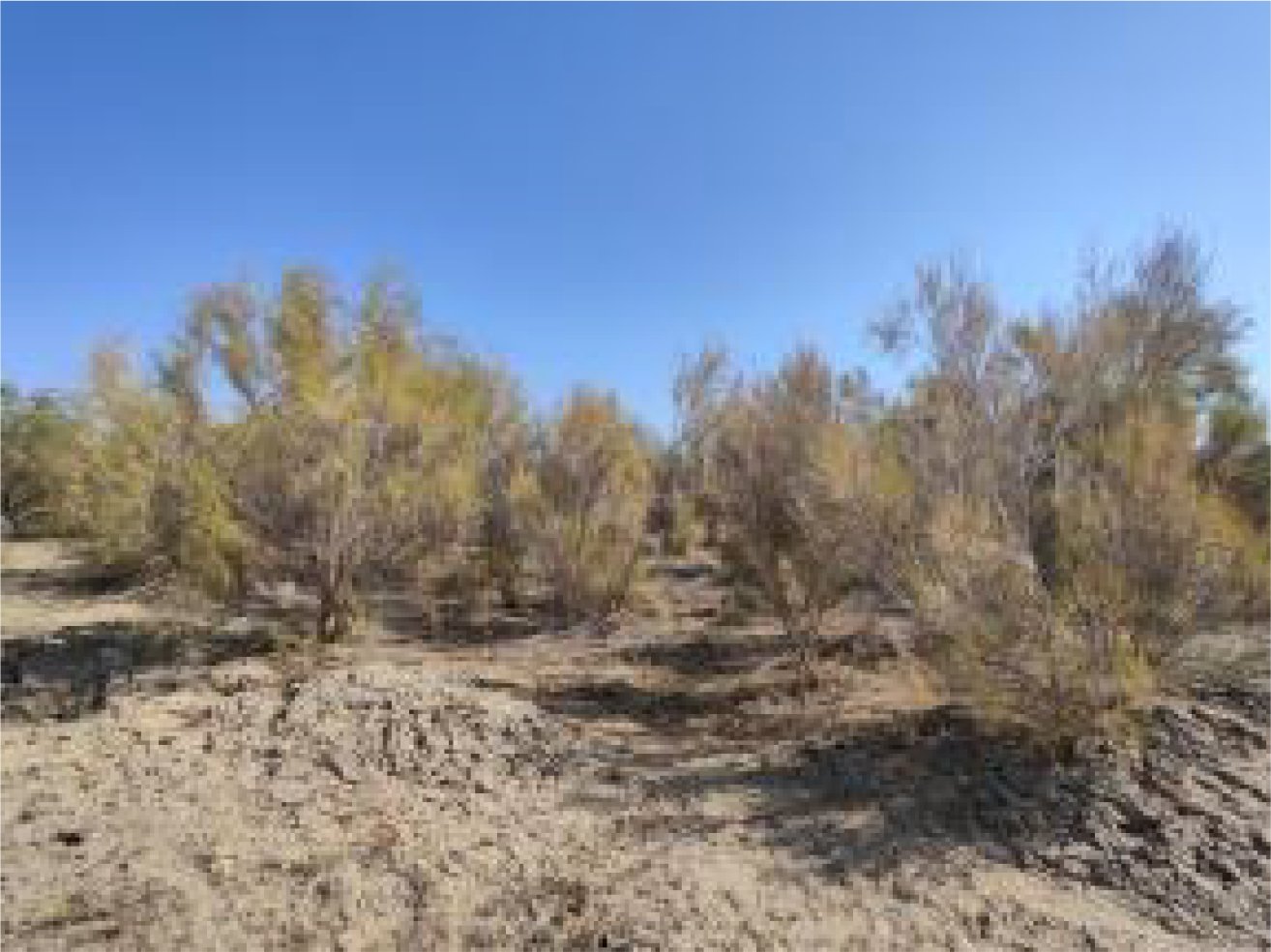

Water from xylem and soil samples was extracted using an automated vacuum-condensation extraction system (LI-2100, Beijing, China) housed in the Key Laboratory of Oasis Ecology, Ministry of Education. The extraction process required approximately 3 h for both xylem and soil samples, with an efficiency exceeding 98% (Yang et al., 2015). After extraction, the water samples were filtered through 0.22 μm pore-size filters and then transferred into 2 mL crimp-cap vials and stored at 4 °C until isotopic analysis. The hydrogen and oxygen stable isotope ratios of the samples were determined using an isotope ratio infrared spectroscopy (IRIS) analyzer – the Liquid Water Isotope Analyzer (TIWA-45-EP, Los Gatos Research Inc., Mountain View, CA, USA) – and expressed in standard delta notation (*δ*²H and *δ*^18^O, ‰) relative to Vienna Standard Mean Ocean Water (V-SMOW) (Equation 1). Considering the hydrogen isotope fractionation in some woody xerophytes (Ellsworth and Williams, 2007), we chose only the *δ*^18^O value as the tracer, which follows:

(1) $$\delta^{18}\text{O}=(\frac{{\text{R}}_{\text{sample}}}{{\text{R}}_{\text{standard}}}-1)\times 1000‰$$

where *R*_sample_ and *R*_standard_ are the oxygen isotopic composition (*δ*^18^O/*δ*^16^O molar ratio) of the sample and the standard water (Standard Mean Ocean Water), respectively. The analytical precision of individual measurements was ±0.25‰ for *δ*^18^O. The oxygen isotopic compositions of plant xylem, soil and groundwater were designated *δ*^18^O_x_, *δ*^18^O_s_, and *δ*^18^O_g_, respectively.

Low-temperature vacuum distillation of plant xylem water can co-distill organic compounds (e.g. methanol and ethanol), which may interfere with spectroscopic analysis and lead to biased isotope values. Therefore, raw plant-water isotope data were corrected using the integrated spectral-correction module in the LWIA Post Analysis software (v. 4.5.0.6) (Schultz et al., 2011).

Methanol spectral pollution correction curve (Equation 2):

(2) $$\Delta \text{δ}18(y)\sim \text{lnNB}(x):\text{y}=0.023452{\text{x}}^{3}+0.005295{\text{x}}^{2}+0.523766\text{x}+1.676226$$

### 2.3 Quantification of the water sources used by the plants

Based on the vertical and seasonal variations in soil water *δ*^18^O values and soil water content, the soil profile was divided into three major depth intervals (Pei et al., 2023). Accordingly, four potential water sources, including groundwater, as following:

1. Shallow soil water (0–40 cm): *δ*^18^O values and soil water content exhibited high variability, being susceptible to rainfall pulses and evaporative processes across seasons.
2. Middle soil water (40–100 cm): compared to the shallow layer, this interval showed lower variability and more subdued monthly fluctuations in both *δ*^18^O values and soil water content.
3. Deep soil water (100–300 cm): *δ*^18^O values and soil water content remained relatively stable, with no significant variations with depth or season.
4. Groundwater: isotopic signals were highly stable owing to minimal evaporative influence.

These four end-members correspond to clear separation between the evaporation-enriched shallow layer, the transitional middle layer, the stable deep layer, and the depleted groundwater end-member. One-way ANOVA confirmed that their water content and isotopic signatures differed significantly from one another (SWC: *F* = 20.42, *P* < 0.05; *δ*^18^O_s_: *F* = 452.48, *P* < 0.05).

### 2.4 Groundwater chemical analysis

Groundwater chemical properties included the concentrations of four major cations (K^+^, Mg^2^+, Na+, and Ca^2+^) and four major anions (CO_3_^2-^, HCO_3_^-^, Cl^-^, and SO_4_^2-^), as well as pH and turbidity as hydrological indicators. Cation concentrations (K^+^, Mg^2^+, Na+, and Ca^2+^) were determined using atomic absorption spectrometry (AAS; PerkinElmer AAnalyst 800, USA). Concentrations of CO_3_^2-^ and HCO_3_^-^ were determined by acid-base titration. Concentrations of Cl^-^ and SO_4_^2-^ were determined by ion chromatography (IC; Thermo Fisher ICS-6000, USA). pH was measured using a pH meter (WTW pH 3310, Germany), and turbidity was measured using a turbidimeter (TU-60, China) (Yan et al., 2023).

### 2.5 Data analysis

The differences in the SWC, *δ*^18^O_s_ and *δ*^18^O_x_ were tested using a linear mixed model with groundwater depth, species, and season as the fixed effects. The differences in the *δ*^18^O_x_ among different seasons or species, and the *δ*^18^O_s_ among different soil layers or end-members were analyzed using one-way analysis of variance (ANOVA) with Tukey’s honestly significant difference (HSD) *post hoc* tests. The relative contribution of the different water sources to the composition of the xylem water was estimated by the IsoSource model (Phillips and Gregg, 2003). The source increment was defined as 2%, and the mass balance tolerance was defined as 0.1‰. Following the standard IsoSource approach, we used the mean value of the feasible source contribution range as the representative value for each water source contribution in subsequent statistical analyses. Linear regression were used to identify the relationship between the use percentage and groundwater depth for *H. ammodendron*. Data analyses were performed using R (version 4.5.0), and the figures were processed using Origin 8.5 software (OriginLab Corp., Northampton, MA, USA).

## 3 Results

### 3.1 Seasonal changes in the soil water content

**Figure 2:**
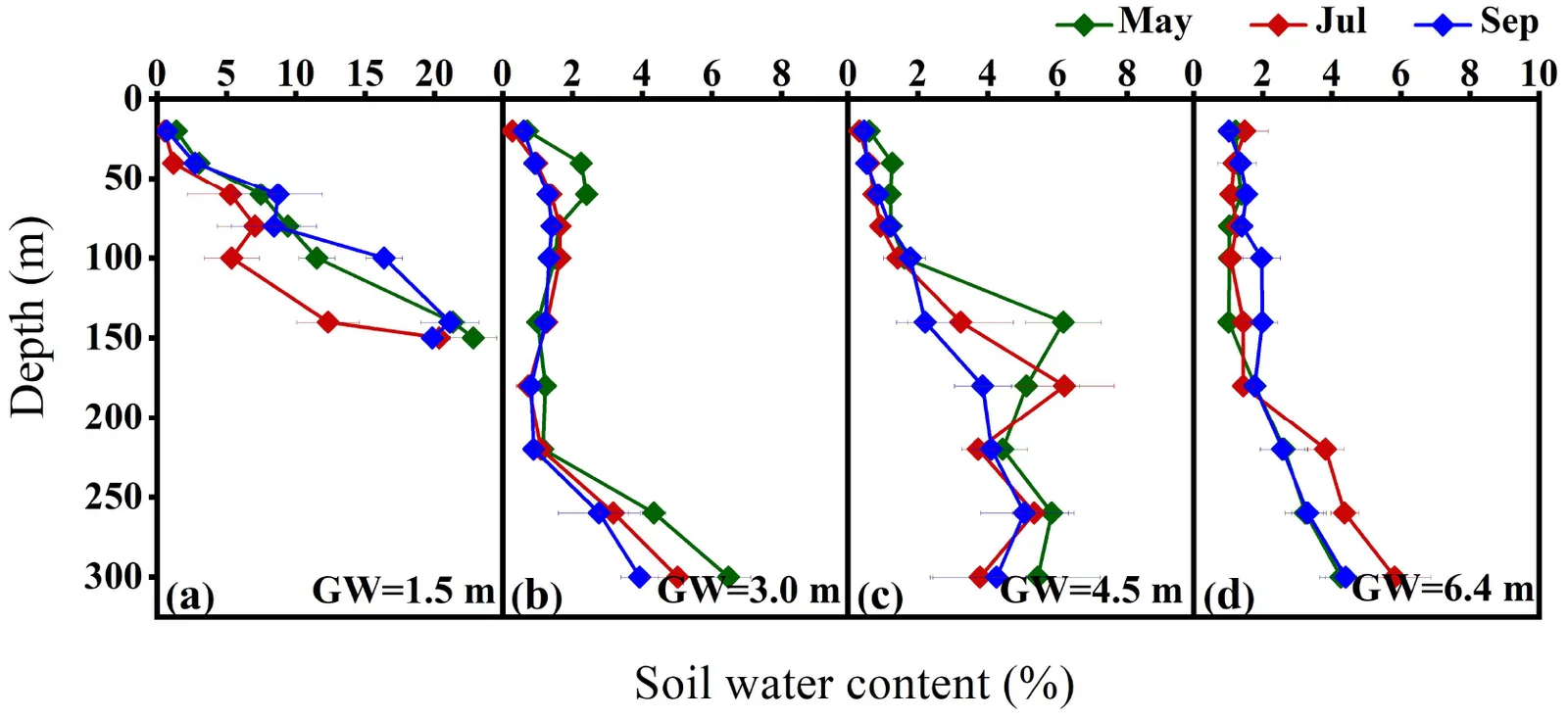
Vertical distribution of the soil water contents along the groundwater depth gradient in the Kalamaili National Park candidate area during the growing season of 2025. Data are presented as the mean ± 1 standard error (*n* = 3). Panels a-d correspond to groundwater depths of 1.5 m, 3.0 m, 4.5 m, and 6.4 m, respectively. Note that x-axis scales differ between diagrams.

SWC exhibited distinct vertical and seasonal variations across the soil profile (**Figure 2**), with significant differences among seasons and groundwater depths (*F* = 3.16, *P* < 0.05; *F* = 116.23, *P* < 0.05). In the upper soil layers (0–100 cm), SWC was generally low and showed strong seasonal fluctuations, characterized by higher values in spring (May) and lower values in summer (July) and early autumn (September). In contrast, SWC in the deep soil layers (below 100 cm) was relatively higher overall. Notably, at the site with a groundwater depth of 1.5 m, SWC in the deep layers was substantially higher than at other sites, reaching a maximum of 25.3%, which markedly exceeded values observed elsewhere.

### 3.2 Seasonal changes in the soil water oxygen isotopic composition

**Figure 3:**
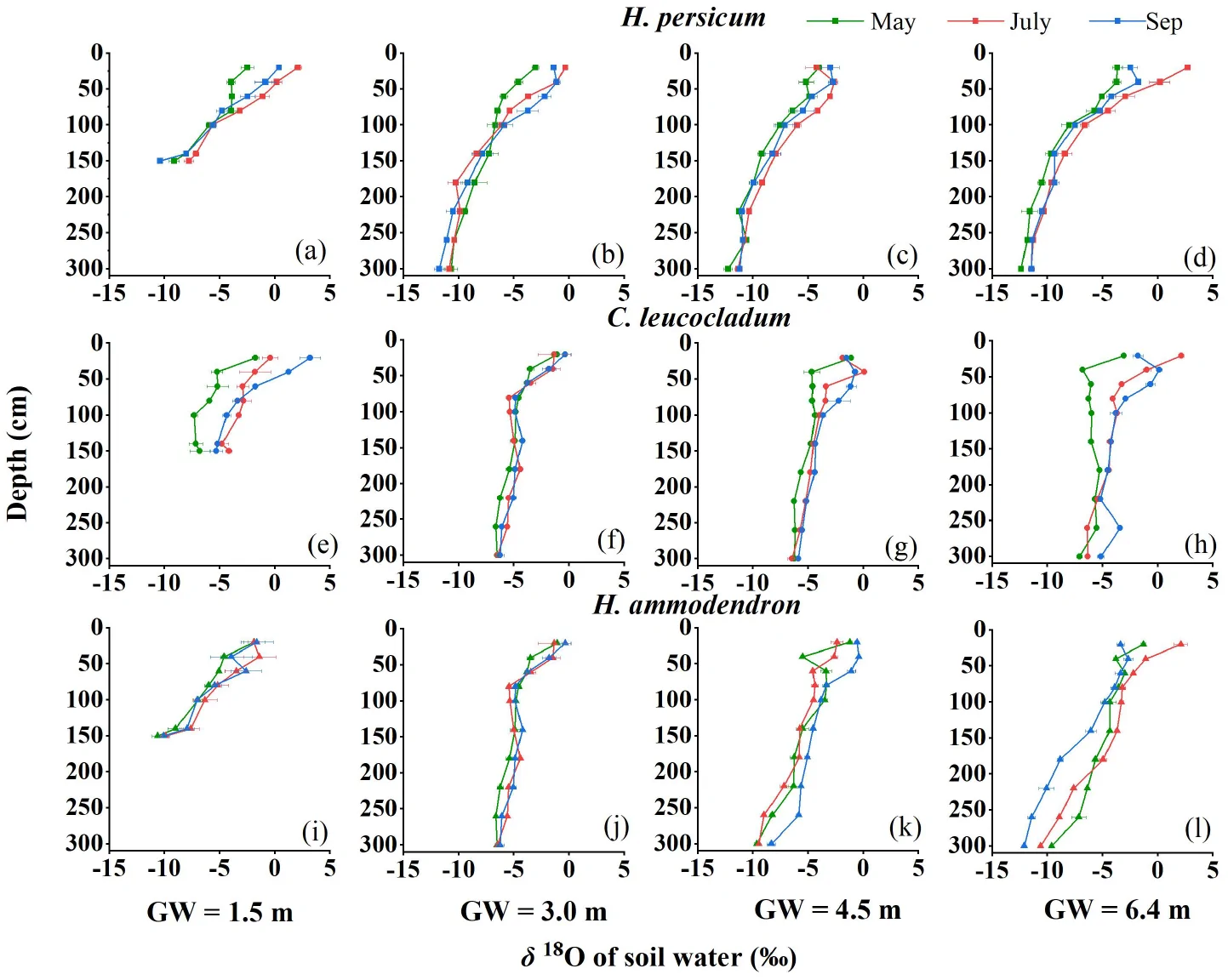
Vertical distribution of soil water oxygen isotopic composition (δ¹⁸O) along the groundwater depth gradient **(a–d)** for H. persicum, **(e–h)** for C. leucocladum, **(i–l)** for H. ammodendron) at four groundwater depths (1.5, 3.0, 4.5, and 6.4 m) during the 2025 growing season. Data are presented as the mean ± 1 standard error (n = 3).

The *δ*^18^O_s_ exhibited significant differences among seasons, species and groundwater depths (*F* = 54.14, *P* < 0.05; *F* = 190.62, *P* < 0.05; *F* = 11.36, *P* < 0.05). As shown in **Figure 3**, the *δ*^18^O_s_ values in the upper soil layers (above 100 cm) exhibited pronounced seasonal variations, especially in the shallow layer (0–40 cm), with more negative values in spring and more positive values in summer and early autumn. In contrast, the *δ*^18^O_s_ values in the deep soil layers (100–300 cm) showed no significant seasonal fluctuations, except for those beneath *H. ammodendron* at GW = 4.5 m and GW = 6.4 m, as well as *C. leucocladum* at GW = 1.5 m and GW = 6.4 m. Moreover, most *δ*^18^O_s_ profiles fluctuated with an overall declining trend, indicating that deep soil water was less enriched in heavy isotopes than shallow soil water. The *δ*^18^O_s_ values in the *H. persicum* habitat were more depleted than those in the habitats of the other two species.

### 3.3 Oxygen isotopic compositions of the xylem water and the potential water sources

**Figure 4:**
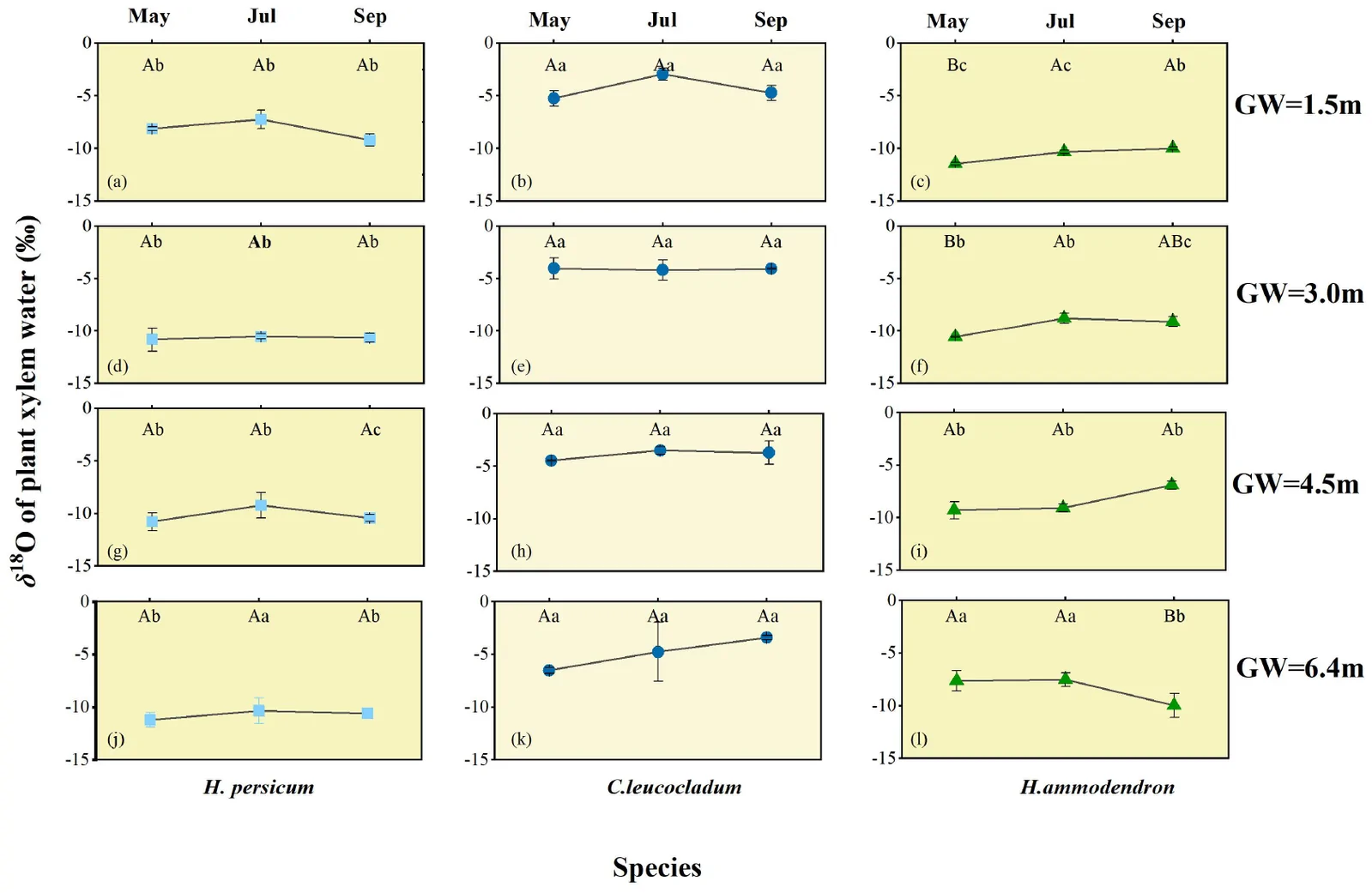
Oxygen isotopic composition of xylem water of *Haloxylon persicum*, *Calligonum leucocladum*, *Haloxylon ammodendron* along the groundwater depth gradient in the Kalamaili National Park candidate area during the growing season of 2025. Panels **(a–c)**, **(d–f)**, **(g–i)**, and **(j–l)** correspond to *H. persicum, C. leucocladum*, and *H. ammodendron* at the 1.5, 3.0, 4.5, and 6.4 m sites, respectively, with measurements from May, July, and September shown in sequence for each species. Different uppercase letters indicate significant differences among seasons, and different lowercase letters indicate significant differences among species. The *δ*^18^O_x_ values are presented as the mean ± 1 standard error (*n* = 3).

The *δ*^18^O_x_ showed significant differences for the effects of season and species (*P* < 0.05), but not for the groundwater depth (**Table 4**). During the growing season, the *δ*^18^O_x_ values of the three studied species exhibited variability, but their seasonal patterns were similar, characterized by more depleted in spring and more enriched in summer and early autumn. These trends particularly evident for *C. leucaclodum* and *H. ammodendron* (**Figure 4**). Compared with *H. persicum and H. ammodendron*, the *δ*^18^O_x_ of *C. leucaclodum* was significant more enriched throughout the entire growing season.

**Table 4:** Summary on the results of linear mixed model on xylem water oxygen isotope composition.

Fixed effects	denDF	*F*	*P*
Season	2	8.11	**<0.01**
Species	2	182.6	**<0.01**
Groundwater depth	3	1.22	0.309
Season*Species	4	0.5	0.738
Groundwater depth*Species	6	0.61	0.719
Season*Groundwater depth	6	5.89	**<0.01**
Season*Species*Groundwater depth	12	2.33	**<0.01**

*P* values less than 0.01 are bold.

### 3.4 Oxygen isotopic compositions of the potential water sources

**Figure 5:**
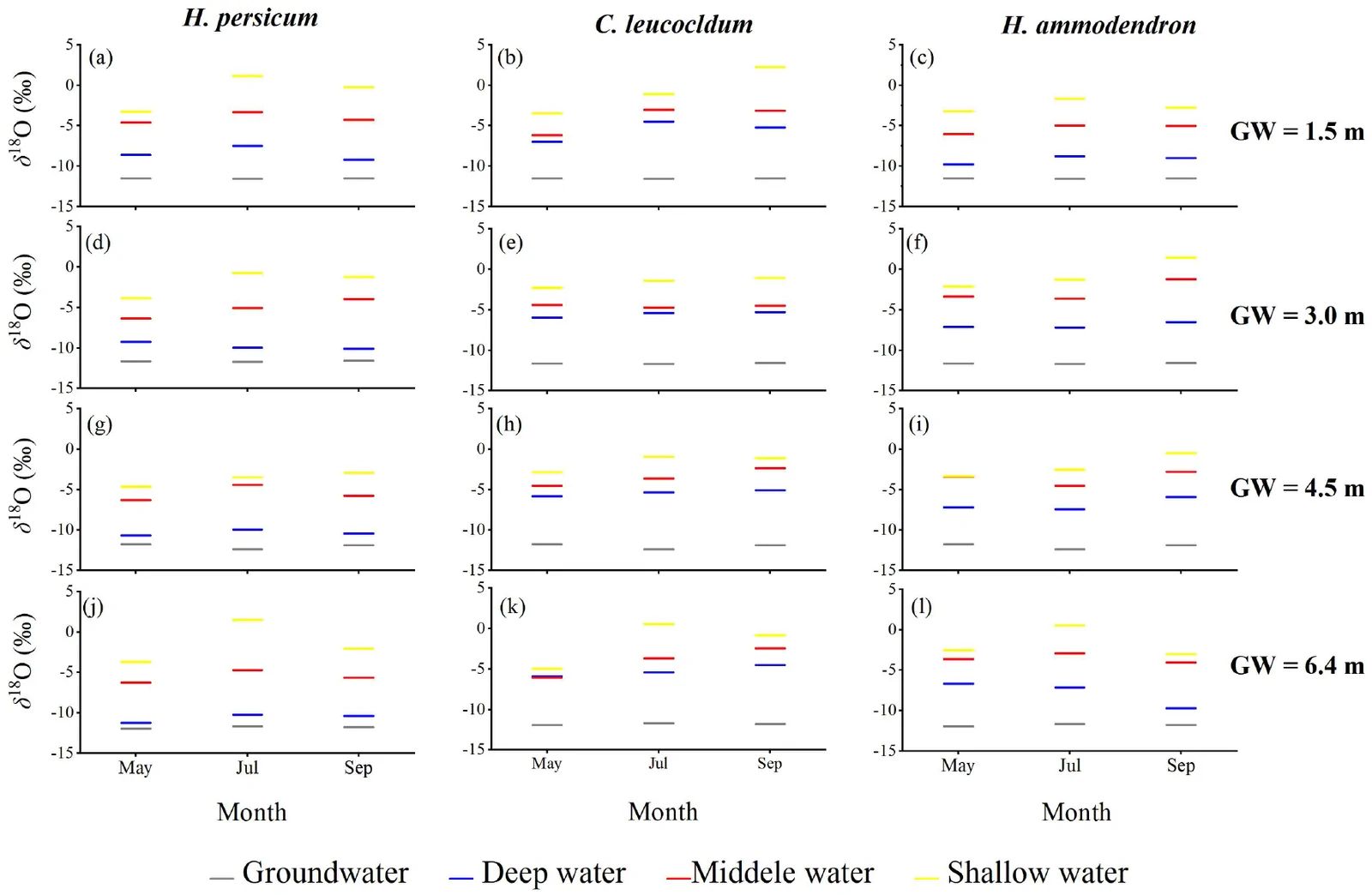
Oxygen isotopic composition of potential water source for *Haloxylon persicum*, *Calligonum leucocladum*, *Haloxylon ammodendron* along the groundwater depth gradient in the Kalamaili National Park candidate area during the growing season of 2025. Panels **(a, d, g, j)**, **(b, e, h, k)**, **(c, f, i, l)** correspond to *H. persicum, C. leucocladum*, and *H. ammodendron* at the 1.5, 3.0, 4.5, and 6.4 m sites, respectively.

Substantial discrepancies in isotopic signatures were observed among the different potential water sources. The *δ*^18^O_g_ just exhibited a little seasonal variation throughout the study period and also showed minor differences among the different groundwater depth sites, ranging from -12.53‰ to -11.47‰ (**Figure 5**). In the shallow soil layers, the *δ*^18^O_s_ displayed an irregular pattern, characterized by marked depletion in spring and pronounced enrichment during dry periods (i.e., summer and autumn) (**Figure 5**). In contrast, the *δ*^18^O_s_ of the middle soil layers only showed slight fluctuations, while those in the deep soil layers exhibited virtually no seasonal variation. Additionally, the *δ*^18^O_s_ of middle and deep soil layers was always more negative for *H. persicum* and *H. ammodendron* than in that of *C. leucocladum*.

### 3.5 Seasonal variations in the water sources along the groundwater depth gradient

**Figure 6:**
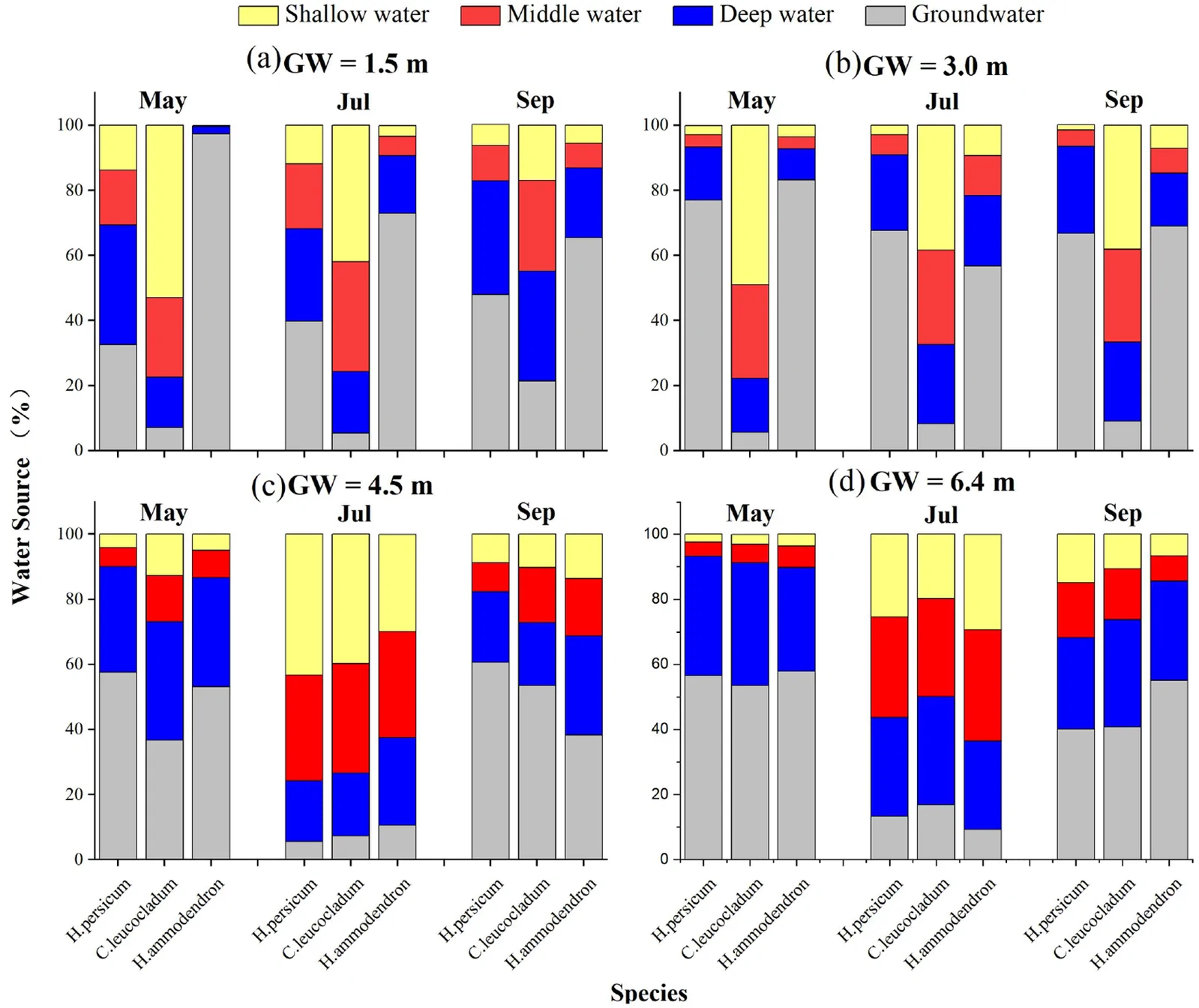
Seasonal variations in the mean percentage of different water sources used by *Haloxylon persicum*, *Calligonum leucocladum*, *Haloxylon ammodendron* along the groundwater depth gradient in the Kalamaili National Park candidate area during the growing season of 2025. **(a–d)** correspond to groundwater depths of 1.5 m, 3.0 m, 4.5 m, and 6.4 m, respectively. The data were obtained from the IsoSource model: shallow soil layer (0–40 cm), middle soil layer (40–100 cm), deep soil layer (100–300 cm) and groundwater.

The mixing models revealed that all three studied species were capable of extracting water from four potential water sources simultaneously; however, the relative contributions of each source varied with season and groundwater depth (**Figure 6**). As shown, groundwater was the predominant water source for *H. ammodendron* throughout the growing season, with average contributions of 72.9% in spring, 37.35% in summer, and 59.9% in autumn. Notably, at the site where the groundwater depth was 1.5 m, the maximum contribution reached 97.3%. *H. persicum* exhibited a similar water uptake pattern across the different study sites, consistently relying on groundwater and deep soil water regardless of variations in groundwater depth, although its dependence on groundwater was lower than that of *H. ammodendron*. In contrast to the two *Haloxylon* species, *C. leucocladum* relied mainly on shallower water sources, with an average contribution of 29% from shallow soil water in spring and 31% from middle soil water in summer.

### 3.6 Relationship between the use percentage of groundwater and groundwater depth

**Figure 7:**
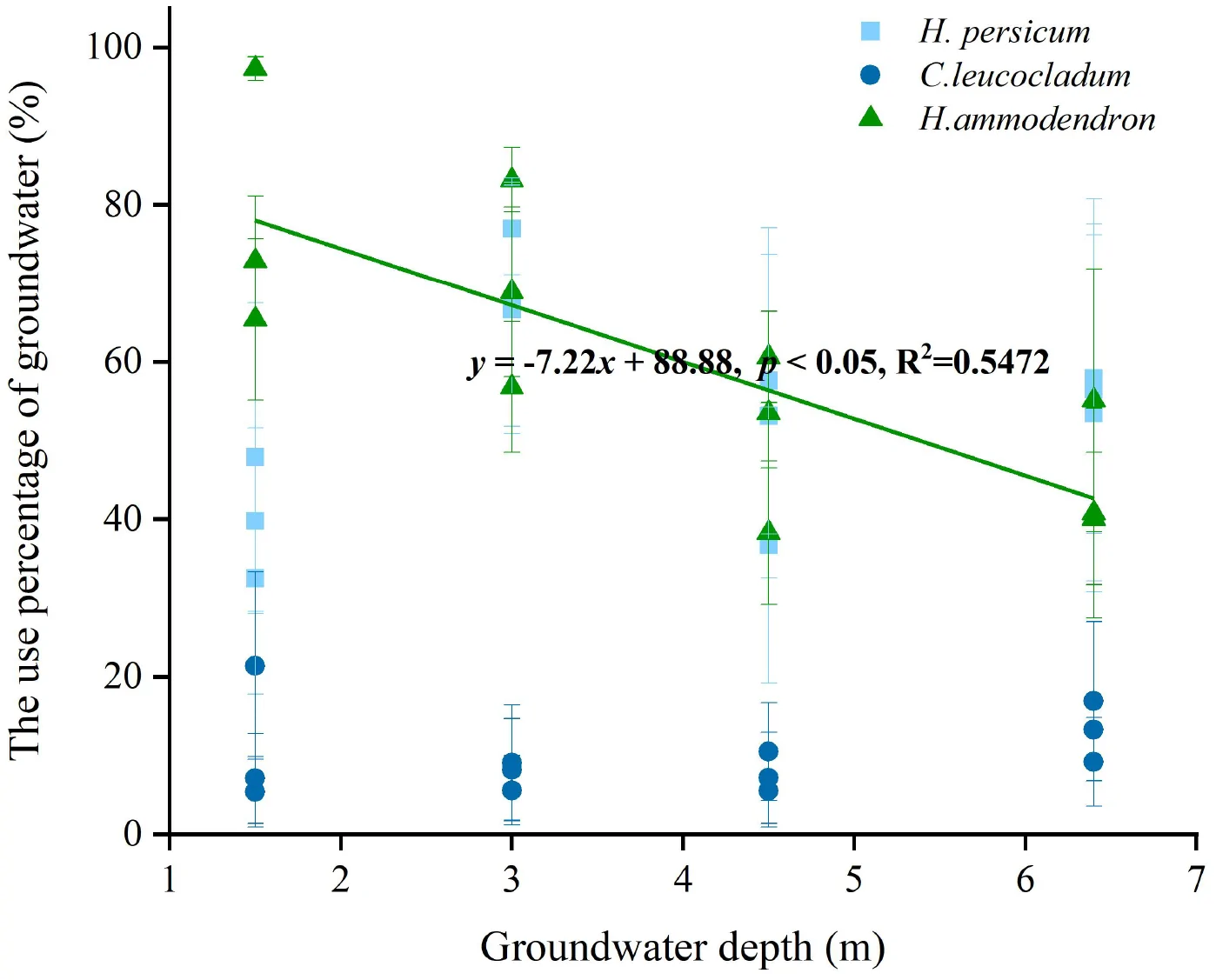
Relationship between the use percentage of groundwater and groundwater depth for *Haloxylon persicum*, *Calligonum leucocladum*, *Haloxylon ammodendron*.

Correlation analysis revealed that only the groundwater use proportion of *H. ammodendron* exhibited a significant negative correlation with groundwater depth (**Figure 7**). A linear regression was subsequently conducted, and the relationship was expressed as follows: *y* = -7.22*x* + 88.88, R² = 0.55 (*P* < 0.05). This finding indicates that groundwater utilization by *H. ammodendron* is adversely impacted by decreasing groundwater accessibility.

## 4 Discussion

### 4.1 Seasonal variations in soil water content and isotopic composition

Soil water stable isotope composition exhibited pronounced seasonal variations, particularly in the upper soil layers (0–100 cm), with more depleted values in spring and progressive enrichment in summer. This seasonal pattern could driven by the interplay of precipitation isotope seasonality, evaporative enrichment, and soil water mixing processes (Zhang et al., 2025).

The observed spring depletion in shallow soil water corresponds to the seasonal characteristics of precipitation isotopes in arid Northwest China, such patterns have been documented in many other locations. For example, in the Manas River Basin, precipitation *δ*^18^O values have been shown to be more depleted in winter and spring and enriched in summer (Lan et al., 2023). Differently, in the Mu Us Sandy Land, precipitation *δ*D and *δ*^18^O values exhibited enrichment in spring and summer and depletion in autumn and winter (Liu et al., 2024). This seasonal isotopic pattern discrepancy in precipitation, which is directly attributed to variations in water vapor sources and local meteorological conditions, influences shallow soil water isotopic signatures as rainfall infiltrates and mixes with existing soil water.

Evaporative enrichment represents another critical factor controlling the seasonal isotopic dynamics of shallow soil water. During summer months, elevated temperatures and increased vapor pressure deficits enhance soil evaporation, causing preferential loss of lighter isotopes and consequent enrichment of ^18^O in the residual soil water (Liu et al., 2024). The pronounced enrichment observed in shallow layers during summer reflects cumulative evaporative effects under high atmospheric evaporative demand. In contrast, spring conditions—characterized by lower temperatures, higher relative humidity, and occasional snowmelt recharge—favor reduced evaporative fractionation and more depleted soil water signatures.

Our soil moisture measurements revealed that shallow-layer (0–40 cm) water content and *δ*^18^O exhibited a coherent reciprocal pattern: moisture was highest in May and September and lowest in July, while *δ*^18^O was most negative in May, most enriched in July, and intermediate in September. This seasonal covariation is consistent with evaporative concentration as the primary dominant seasonal driver—enhanced evaporative demand during the hot, dry summer simultaneously reduces soil moisture and enriches the residual water in ^18^O, while reduced evaporation during cooler transitional months allows moisture accumulation and isotopic depletion.

The stable isotopic composition of deep soil water (100–300 cm) throughout the seasons indicates that deeper layers are buffered from direct evaporative effects and seasonal precipitation pulses (Wang et al., 2017). Similar findings have been reported for desert ecosystems, where deep soil water isotopic signatures approach those of groundwater and show minimal seasonal variation due to limited water mixing and the predominance of piston flow transport mechanisms (Dai et al., 2015). Importantly, this isotopic stability was complemented by sustained moisture availability: deep-layer soil water content remained consistently higher and exhibited markedly lower seasonal variability. This combined evidence—isotopic invariance coupled with stable water content—supports the inference that the deep soil layer functions as a buffered water reservoir during drought periods, when shallow soil moisture is depleted. Nevertheless, it is necessary to employ sap-flow or soil matric potential sensors provide more direct validation of deep water availability to plants in future studies.

### 4.2 Species-specific differences in water use strategies among desert plants

The three studied species exhibited markedly different water uptake strategies, reflecting distinct morphological and physiological adaptations to the heterogeneous water availability conditions in the Kalamaili region. *H. ammodendron* demonstrated the highest reliance on groundwater throughout the growing season. Root excavation experiments confirmed that *H. ammodendron* possesses a stout, robust, and deep-reaching taproot system—with sparse lateral roots and a narrow root spread—capable of extending to depths exceeding 10 m (Shen et al., 2004), which enables direct access to the stable groundwater table and explains its strong dependence on groundwater. Such a deep-rooting strategy allows *H. ammodendron* to bypass the seasonally fluctuating soil water zone and maintain a consistent water supply even during drought periods. This finding is consistent with previous studies in the Gurbantonggut Desert, where *H. ammodendron* was shown to predominantly utilize deep soil water and groundwater (Dai et al., 2015; Liu et al., 2018; Tiemuerbieke et al., 2018).

In contrast, *C. leucocladum* relied predominantly on shallower water sources, which is consistent with its root architecture. Previous studies have shown that *C. leucocladum* possesses extensive horizontal roots predominantly distributed in the 10–20 cm shallow sand layer, with lateral roots extending radially up to 20 m (Zhang et al., 2014). This morphological adaptation enables efficient capture of moisture from light precipitation events and surface soil water, but limits access to deeper groundwater. Such a strategy is typical of psammophytic shrubs that stabilize mobile sand dunes through extensive lateral root networks (Xu et al., 2016). *Haloxylon persicum* exhibited an intermediate water use strategy, primarily relying on deep soil water rather than direct groundwater uptake. This pattern may be attributed to both rooting morphology and potential salt tolerance constraints, although our current dataset does not permit quantitative partitioning of their relative contributions. Root excavation experiments demonstrated that *H. persicum* develops deep root systems capable of extending to depths exceeding 18 m. The majority of fine roots are concentrated within the upper 6 m of the profile; however, at sites with greater groundwater depth, fine root distribution shifts downward, accompanied by a reduction in fine root abundance near the soil surface (Xu et al., 2017). Unlike *H. ammodendron*, which typically occupies interdune lowlands with relatively shallow groundwater tables, *H. persicum* is typically located on the upper portions of sand dunes, where the groundwater table is considerably deeper and less accessible. This topographic position therefore limits its access to the permanent groundwater pool (Dai et al., 2015). In addition, the lower reliance on groundwater in *H. persicum* may also be attributed to its relatively poor tolerance to salinity compared to *H. ammodendron*. Previous studies have demonstrated that under high NaCl concentrations (e.g., 700 mmol/L), *H. persicum* exhibits significantly lower K^+^ content and K^+^/Na^+^ ratios in its embryos, as well as greater membrane damage, indicating weaker salt resistance (Song et al., 2009). Given the high salinity of groundwater in the Kalamaili region (Na^+^concentration up to 4777.74 mg/L), direct groundwater uptake may impose osmotic and ionic stress on *H. persicum*, further constraining its utilization of this stable but saline water source. Consequently, *H. persicum* depends more on deep soil water recharged by occasional precipitation or lateral flow, rather than on the permanent groundwater table. This water use strategy, shaped by both rooting morphology and physiological salt limitation, appears to represent a long-term adaptation to the unique soil-water environment of the Kalamaili region. However, the lack of synchronous measurements of groundwater salinity at the specific rooting zones of the sampled individuals precludes confirmation of above speculation, which remains to be tested in future studies.

In our study, three species employed different water source. The coexistence of these three species with contrasting water use strategies within the same habitat exemplifies hydrological niche separation, a key mechanism for reducing interspecific competition in water-limited ecosystems (Muñoz-Gálvez et al., 2025). As demonstrated in the Badain Jaran Desert, coexisting shrubs with different root biomass distributions in shallow versus deep soil layers exhibited distinct water source dependencies, thereby minimizing resource competition (Song et al., 2025). This functional diversity in water acquisition not only reduces interspecific competition but also enhances ecosystem resilience to seasonal and interannual climate variability (Fan et al., 2025).

### 4.3 Relationships between groundwater depth and plant water use

Groundwater depth is a critical factor regulating water availability and shaping water use strategies (Antunes et al., 2018). In the Kalamaili region, the negative response of groundwater use proportion by *H. ammodendron* to increasing groundwater depth indicates that deeper water tables restrict the accessibility of this stable water source. As groundwater depth increases, the proportional contribution of groundwater to total plant water uptake declines, forcing plants to rely more on alternative sources such as deep soil water. Previous research has documented that *H. ammodendron* can derive a substantial proportion of its water from groundwater when the water table is shallow, but this contribution decreases markedly when groundwater depth exceeds critical thresholds (Wu et al., 2019a). The observed groundwater depth threshold for significant reduction in groundwater use proportion by deep-rooted species in this study is consistent with findings from other arid regions, although the absolute threshold values vary across sites due to differences in species, climate, and hydrogeological conditions (Chen et al., 2026).

Beyond this general pattern, the three species exhibited differential shifts in water source partitioning in response to groundwater depth variation, reflecting species-specific hydrological niche differentiation. Unlike *H. ammodendron*, *H. persicum* maintained a relatively stable reliance on both groundwater and deep soil water across varying groundwater depths, suggesting a flexible source-switching strategy. In contrast, *C. leucocladum* relied predominantly on shallow and middle soil water regardless of groundwater depth, indicating that its water use is largely decoupled from groundwater table fluctuations. These contrasting strategies imply that the three species will respond differently to ongoing hydrological changes. Projected declines in groundwater tables due to increased water extraction and climate change may fundamentally alter water source partitioning in groundwater-dependent ecosystems (Eamus et al., 2006). From a hydrological niche perspective, these three strategies will shape contrasting population survival potentials under declining groundwater and changing precipitation regimes. For *H. ammodendron*, continued groundwater decline directly threatens its primary water source, and its limited capacity to shift to shallower sources makes it the most vulnerable species (Wu et al., 2019b). For *H. persicum*, the flexible switching between groundwater and deep soil water may confer greater hydrological resilience, although its poor salt tolerance may constrain groundwater use as salinity increases with declining water tables. For *C. leucocladum*, population survival depends more on precipitation patterns; its shallow-rooted strategy would face severe stress under reduced or more variable rainfall, but its extensive lateral roots may provide an advantage in capturing episodic rainfall events. In summary, the contrasting hydrological niches —groundwater-dependent (*H. ammodendron*), flexible two-source (*H. persicum*), and shallow-soil-dependent (*C. leucocladum*)—will determine their differential responses to ongoing hydrological changes in the Kalamaili region.

### 4.4 Study limitations and future directions

The above predictions regarding species-specific responses to hydrological changes should be interpreted with caution, given several limitations of this study. First, although sampling was conducted in key phenological nodes to capture growing-season dynamics, our temporal resolution remains limited to three discrete time points (May, July, and September), which cannot resolve sub-daily or synoptic-scale plant water uptake dynamics between sampling dates. Consequently, long-term multi-year monitoring would be required to test whether these short-term dynamics eventually translate into stable coexistence. Second, soil sampling did not reach the groundwater table at deeper sites (≥ 3.0 m) due to the difficulty of excavating in loose aeolian sands; the unsampled interval between the lowest sampling depth and the water table may contain additional water sources and influence source partitioning results. Although isotopic gradients in deep vadose zones are typically gradual and evaporation effects are negligible below 1 m, this gap should be considered when interpreting our quantitative estimates. Finally, despite conducting all sampling after multiple consecutive rainless days, the legacy effects of earlier rainfall events cannot be completely excluded.

Despite these limitations, this study provides valuable insights into the hydrological niche differentiation and water use strategies of coexisting desert plants in the Kalamaili region. Future studies with long-term, multi-seasonal monitoring, higher temporal frequency (e.g., *in-situ* sap flow or soil-plant-atmosphere continuum sensors to capture diurnal dynamics), deeper soil sampling (down to water table), and multi-site comparative frameworks across contrasting groundwater gradients and community assemblages are needed to constrain uncertainties and improve understanding of ecohydrological processes in this arid region under changing environmental conditions.

## 5 Conclusions

This study employed stable oxygen isotope techniques to reveal water use strategies of three coexisting desert species (*Haloxylon ammodendron*, *Haloxylon persicum*, and *Calligonum leucocladum*) in the Kalamaili National Park candidate. Our results reveal clear hydrological niche partitioning among the three species, with differentiated reliance on groundwater, deep soil water, and shallow-to-middle soil water, respectively, suggesting a mechanism that may reduce interspecific competition. Groundwater use by *H. ammodendron* exhibited a significant negative correlation with increasing groundwater depth, while *H. persicum* displayed high water source plasticity and *C. leucocladum* was largely decoupled from groundwater. Based on these patterns, we propose the “asymmetric groundwater sensitivity” as a mechanistic for transient coexistence or short-term niche differentiation during the growing season among desert woody species: differential responsiveness to water-table fluctuation may generate complementary resource use and temporarily reduce interspecific competition. The patterns observed in our study represent a site-specific manifestation and require future multi-site validation. Within the acknowledged limitations, our findings provide a conceptual basis for understanding hydrological niche differentiation in groundwater-dependent arid ecosystems and guiding adaptive management in arid regions.

## Data Availability

The raw data supporting the conclusions of this article will be made available by the authors, without undue reservation.

## References

[B1] Antunes C. Chozas S. West J. Zunzunegui M. Diaz Barradas M. C. Vieira S. . (2018). Groundwater drawdown drives ecophysiological adjustments of woody vegetation in a semi-arid coastal ecosystem. Global Change Biol. 24, 4894–4907. doi: 10.1111/gcb.14403 30030867

[B2] Barbeta A. Burlett R. Martín-Gómez P. Fréjaville B. Devert N. Wingate L. . (2022). Evidence for distinct isotopic compositions of sap and tissue water in tree stems: consequences for plant water. New Phytol. 233, 1121–1132. doi: 10.1111/nph.17857 34767646

[B3] Broekman M. J. Muller-Landau H. C. Visser M. D. Jongejans E. Wright S. J. de Kroon H. (2019). Signs of stabilisation and stable coexistence. Ecol. Lett. 22, 1957–1975. doi: 10.1111/ele.13349 31328414

[B4] Cai L. Xiong K. Li Y. Liu Z. Q. Zhu D. Y. Liang H. . (2025). Coexisting plants restored in karst desertification areas cope with drought by changing water uptake patterns and improving water use efficiency. J. Hydrol. 654, 132813. doi: 10.1016/j.jhydrol.2025.132813 42574925

[B5] Cao Y. S. Xiao B. Sun F. H. Heitman J. (2025). Enriched stable hydrogen and oxygen isotopes in biocrusts unveil their critical roles in mediating ecohydrological processes of drylands. Plant Soil 514, 1903–1921. doi: 10.1007/s11104-025-07497-1 30311153

[B6] Chen C. Yuan K. Y. Ling H. B. (2026). Relationship between community characteristics of desert plants and ecological threshold of groundwater depth. Arid Zone Res. 43, 350–360. doi: 10.13866/j.azr.2026.02.12

[B7] Chesson P. Gebauer R. L. E. Schwinning S. Huntly N. Wiegand K. Ernest M. S. K. . (2004). Resource pulses, species interactions, and diversity maintenance in arid and semi-arid environments. Oecologia 141, 236–253. doi: 10.1007/s00442-004-1551-1 15069635

[B8] Dai Y. Zheng X. J. Tang L. S. Li Y. (2015). Stable oxygen isotopes reveal distinct water use patterns of two Haloxylon species in the Gurbantonggut Desert. Plant Soil 389, 73–87. doi: 10.1007/s11104-014-2342-z 30311153

[B9] Dawson T. E. Mambelli S. Plamboeck A. H. Templer P. H. Tu K. P. (2002). Stable isotopes in plant ecology. Annu. Rev. Ecol. Systematics 33, 507–559. doi: 10.1146/annurev.ecolsys.33.020602.095451 41139587

[B10] de la Casa J. Barbeta A. Rodríguez-Uña A. Wingate L. Ogée J. Gimeno T. E. (2021). Revealing a significant isotopic offset between plant water and its sources using a global meta-analysis. Hydrol. Earth Syst. Sci. 25, 5097–5110. doi: 10.5194/hess-2021-333

[B11] Eamus D. Froend R. Loomes R. Hose G. Murray B. (2006). A functional methodology for determining the groundwater regime needed to maintain the health of groundwater-dependent vegetation. Aust. J. Bot. 54, 97–114. doi: 10.1071/bt05031 38477348

[B12] Eggemeyer K. D. Awada T. Harvey F. E. Wedin D. A. Zhou X. H. Zanner C. W. (2009). Seasonal changes in depth of water uptake for encroaching trees Juniperus virginiana and Pinus ponderosa and two dominant C4 grasses in a semiarid grassland. Tree Physiol. 29, 157–169. doi: 10.1093/treephys/tpn019 19203941

[B13] Eheringer J. R. Dawson T. E. (1992). Water uptake by plants: perspectives from stable isotope composition. Plant Cell Environ. 15, 1073–1082. doi: 10.1111/j.1365-3040.1992.tb01657.x

[B14] Ellsworth P. Z. Williams D. G. (2007). Hydrogen isotope fractionation during water uptake by woody xerophytes. Plant Soil 291, 93–107. doi: 10.1007/s11104-006-9177-1 30311153

[B15] Fan M. Y. Zhou H. Tian L. H. Ren H. Liu B. Ji X. B. . (2025). Hydrological niche separation between two coexisting shrubs in an extremely arid region. J. Hydrol.: Regional Stud. 57, 102170. doi: 10.1016/j.ejrh.2024.102170 42574925

[B16] Fatichi S. Leuzinger S. Paschalis A. Langley J. A. Barraclough A. D. Hovenden M. J. (2016). Partitioning direct and indirect effects reveals the response of water-limited ecosystems to elevated CO2. Proc. Natl. Acad. Sci. 113, 12757–12762. doi: 10.1073/pnas.1605036113 27791074 PMC5111654

[B17] Gu H. L. Zhou H. Chen G. P. Ren H. Liu B. Ji X. B. . (2025). Isotopic evidence of hydrological niche segregation and species coexisting mechanism of desert shrubs *Reaumuria songarica* and *Nitraria sphaerocarpa*. J. Hydrol. 661, 133803. doi: 10.1016/j.jhydrol.2025.133803 42574925

[B18] Guan J. Y. Sun Z. Y. Xu X. L. Yang J. J. Liu X. Y. Wang M. C. (2025). Research on the trade-offs and synergy between ecological and social values in the creation area of the Kalamali National Park. Acta Ecol. Sin. 45, 3006–3020. doi: 10.20103/j.stxb.202407011523

[B19] Hardin G. (1960). The competitive exclusion principle: an idea that took a century to be born has implications in ecology, economics, and genetics. Science 131, 1292–1297. 14399717 10.1126/science.131.3409.1292

[B20] Hu Z. Y. Dai Q. H. Li H. Y. Yan Y. L. Zhang Y. Yang X. . (2024). Response of ecosystem water-use efficiency to global vegetation greening. Catena 239, 107952. doi: 10.1016/j.catena.2024.107952 42574925

[B21] Lan X. Jia W. X. Zhu G. F. Zhang Y. Yu Z. J. Luo J. F. (2023). Spatial and temporal variation characteristics of stable isotopes in precipitation and their relationships with meteorological factors in the Shiyang River Basin in China. Water 15, 3836. doi: 10.3390/w15213836 30654563

[B22] Lei Z. R. Jia G. D. Yu X. X. Liu Z. H. (2022). A review of stable hydrogen and oxygen isotopic offset in plant water source research. Chin. J. Plant Ecol. 47, 25–40. doi: 10.17521/cjpe.2021.0479

[B23] Li J. C. Shao C. L. Gao S. S. Li J. (2025a). Summer water source utilization patterns, activity range and suitable habitat distribution of Mongolian wild ass (*Equus hemionus*) in candidate area of Xinjiang's Kalamaili National Park. Biodivers. Sci. 31, 2993–3004.

[B24] Li J. C. Wu P. F. Xing W. Y. Liang F. C. Tian Y. J. Zhu Y. J. . (2025b). Changes in ecological quality of the vegetations and identification of priority restoration areas in Kalamaili National Park candidate area. Acta Ecol. Sin. 45, 9840–9851. doi: 10.20103/j.stxb.202411152799

[B25] Liang C. Z. Zhang M. Y. Wang Z. Xiang X. Q. Gong H. B. Wang K. L. . (2024). The strengthened impact of water availability at interannual and decadal time scales on vegetation GPP. Global Change Biol. 30, e17138. doi: 10.1111/gcb.17138 38273499

[B26] Liu B. Guan H. D. Zhao W. Z. Yang Y. T. Li S. B. (2017). Groundwater facilitated water-use efficiency along a gradient of groundwater depth in arid northwestern China. Agric. For. Meteorol. 233, 235–241. doi: 10.1016/j.agrformet.2016.12.003 42574925

[B27] Liu J. M. Si Z. Y. Li S. Abubakar S. A. Zhang Y. Y. Wu L. F. . (2023). Three Bayesian tracer models: which is better for determining sources of root water uptake based on stable isotopes under various soil water conditions? Agronomy 13, 843. doi: 10.3390/agronomy13030843 30654563

[B28] Liu X. H. Tian S. L. Ma Y. D. He Q. Shi C. C. Zheng C. (2024). Isotopic characteristics and migration of water vapor in the vadose zone of Mu Us Sandy Land. Trans. Chin. Soc. Agric. Eng. 40, 112–120.

[B29] Liu R. Wang Y. G. Li C. J. Ma J. Li Y. (2018). Partitioning water source and sinking process of a groundwater-dependent desert plant community. Plant Soil 430, 73–85. doi: 10.1007/s11104-018-3714-6 30311153

[B30] Maestre F. T. Guirado E. Armenteras D. Beck H. E. Alshalan M. S. Saud N. T. A. . (2025). Bending the curve of land degradation to achieve global environmental goals. Nature 644, 347–355. doi: 10.1038/s41586-025-09365-5 40804148

[B31] Morgan B. E. Araki R. Trugman A. T. Caylor K. K. (2025). Ecological and hydroclimatic determinants of vegetation water-use strategies. Nat. Ecol. Evol. 9, 1791–1799. doi: 10.5194/egusphere-egu25-14060 40730650

[B32] Muñoz-Gálvez F. J. Querejeta J. I. Moreno-Gutiérrez C. Ren W. de la Riva E. G. Prieto I. (2025). Trait coordination and trade-offs constrain the diversity of water use strategies in Mediterranean woody plants. Nat. Commun. 16, 4103. doi: 10.1038/S41467-025-59348-3 40316526 PMC12048502

[B33] Nan Y. C. Huo J. Q. Han G. L. Hu R. Zhao Y. Zhang Y. F. . (2025). Groundwater altered water balance and plant water use efficiency in desert ecosystems. Water Resour. Res. 61, e2025WR040545. doi: 10.1029/2025wr040545 29143083

[B34] Nie Y. P. Chen H. S. Wang K. L. Tan W. Deng P. Y. Yang J. (2011). Seasonal water use patterns of woody species growing on the continuous dolostone outcrops and nearby thin soils in subtropical China. Plant Soil 341, 399–412. doi: 10.1007/s11104-010-0653-2 30311153

[B35] Pei Y. W. Huang L. M. Shao M. A. Jia X. X. Tang X. Z. Zhao Y. L. . (2023). Water sources used by artificial Salix psammophila in stands of different ages based on stable isotope analysis in northeastern Mu Us Sandy Land. Catena 226, 107087. doi: 10.1016/j.catena.2023.107087 42574925

[B36] Pérez-Ramos I. M. Matías L. Gómez-Aparicio L. Godoy O. (2019). Functional traits and phenotypic plasticity modulate species coexistence across contrasting climatic conditions. Nat. Commun. 10, 2555. doi: 10.1038/s41467-019-10453-0 31186418 PMC6560116

[B37] Phillips D. L. Gregg J. W. (2003). Source partitioning using stable isotopes: coping with too many sources. Oecologia 136, 261–269. doi: 10.1007/s00442-003-1218-3 12759813

[B38] Rong L. L. Wang Y. Meidl P. Baqar M. Li A. D. Wang L. . (2025). Insights into soil microbial assemblages and nitrogen cycling function responses to conventional and biodegradable microplastics. J. Hazard. Mater. 491, 137889. doi: 10.1016/j.jhazmat.2025.137889 40081053

[B39] Ruehr S. Girotto M. Verfaillie J. G. Baldocchi D. Cabon A. T. Keenan T. (2023). Ecosystem groundwater use enhances carbon assimilation and tree growth in a semi-arid Oak Savanna. Agric. For. Meteorol. 342, 109725. doi: 10.1016/j.agrformet.2023.109725 42574925

[B40] Sardans J. Miralles A. Tariq A. Zeng F. J. Wang R. Josep P. (2024). Growing aridity poses threats to global land surface. Commun. Earth Environ. 5, 776. doi: 10.1038/s43247-024-01935-1 37880705

[B41] Schultz N. M. Griffis T. J. Lee X. Baker J. M. (2011). Identification and correction of spectral contamination in 2H/1H and 18O/16O measured in leaf, stem, and soil water. Rapid Commun. Mass Spectrom. 25, 3360–3368. doi: 10.1002/rcm.5236 22006400

[B42] Shao C. L. Gao S. S. Ayiejiang Y. Li J. (2025). Study on water resource utilization patterns and suitable habitats assessment of *Goitered gazelle* during the warm season in the Kalamaili National Park (candidate area), Xinjiang. Natural Protected Areas 5, 52–61.

[B43] Shen J. H. Qiao Y. X. Liu H. Y. Zhai Z. X. Guo Y. M. (2004). A study on the root system of *Haloxylon ammodendron* (C. A. Mey.) Bunge. Acta Agrestia Sin. 2, 91–94.

[B44] Shi H. B. Shi Q. D. Li H. Zhou X. L. Dai Y. Kahaer Y. . (2022). The combined effect of surface water and groundwater on environmental heterogeneity reveals the basis of beta diversity pattern in desert oasis communities. PloS One 17, e0279704. doi: 10.1371/journal.pone.0279704 36574442 PMC9794059

[B45] Silvertown J. (2004). Plant coexistence and the niche. Trends Ecol. Evol. 19, 605–611. doi: 10.1016/j.tree.2004.09.003 42574925

[B46] Silvertown J. Araya Y. N. Gowing D. (2015). Hydrological niches in terrestrial plant communities: a review. J. Ecol. 103 (1), 93–108.

[B47] Song J. Tian C. Y. Zhang L. Y. Feng G. (2009). Effect of NaCl on salt resistance of *Suaeda physophora*, *Haloxylon ammodendron* and *Haloxylon persicum* during their seed germination and young seedling stages. Arid Zone Res. 26 (4), 543–547. doi: 10.13866/j.azr.2009.04.023

[B48] Song K. C. Hu H. Y. Zhang H. Guan S. Y. Deng W. H. Yong J. Y. . (2025). Spatiotemporal niche separation mechanisms of water utilization strategies in the desert steppe plant communities, northern China. J. Arid. Land 17, 1741–1760. doi: 10.1007/s40333-025-0034-6 30311153

[B49] Song L. Yang B. Liu L. L. Mo Y. X. Liu W. J. Meng X. J. . (2022). Spatial-temporal differentiations in water use of coexisting trees from a subtropical evergreen broadleaved forest in Southwest China. Agric. For. Meteorol. 316, 108862. doi: 10.1016/j.agrformet.2022.108862 42574925

[B50] Tiemuerbieke B. Min X. J. Zang Y. X. Xing P. Ma J. Y. Sun W. (2018). Water use patterns of co-occurring C_3_ and C_4_ shrubs in the Gurbantonggut desert in northwestern China. Sci. Total Environ. 634, 341–354. 29627558 10.1016/j.scitotenv.2018.03.307

[B51] Wang J. Fu B. Lu N. Zhang L. (2017). Seasonal variation in water uptake patterns of three plant species based on stable isotopes in the semi-arid Loess Plateau. Sci. Total Environ. 609, 27–37. doi: 10.1016/j.scitotenv.2017.07.133 28734247

[B52] Wang Y. Li W. Z. Cai W. Bai N. Wang J. Q. Yu H. (2026). Seasonal shifts in water utilization strategies of typical desert plants in a desert oasis revealed by hydrogen and oxygen stable isotopes and leaf δ¹³C. Plants 15, 340. doi: 10.3390/plants15020340 41600147 PMC12845462

[B53] Wang T. Y. Wu Z. N. Wang P. Wu T. H. Zhang Y. C. Yin J. . (2023). Plant-groundwater interactions in drylands: A review of current research and future perspectives. Agric. For. Meteorol. 341, 109636. doi: 10.1016/j.agrformet.2023.109636 42574925

[B54] Wu X. Zheng X. J. Yin X. W. Yue Y. M. Liu R. Xu G. Q. . (2019b). Varying responses of two Haloxylon species to extreme drought and groundwater depth. Environ. Exp. Bot. 158, 63–72. doi: 10.1016/j.envexpbot.2018.11.014 42574925

[B55] Wu X. Zheng X. J. Xu G. Q. Li Y. (2019a). Seasonal variation in the groundwater dependency of two dominant woody species in a desert region of Central Asia. Plant Soil 444, 27–40. doi: 10.1007/s11104-019-04251-2 30311153

[B56] Xu S. Q. Ji X. B. Jin B. W. Zhang J. L. (2016). Root distribution of three dominant desert shrubs and their water uptake dynamics. J. Plant Ecol. 10, 780–790. doi: 10.1093/jpe/rtw079

[B57] Xu G. Q. Yu D. D. Li Y. (2017). Patterns of biomass allocation in *Haloxylon persicum* woodlands and their understory herbaceous layer along a groundwater depth gradient. For. Ecol. Manage. 395, 37–47. doi: 10.1038/srep15997 26525117 PMC4630587

[B58] Xu X. Zhao Z. G. Skrzypek G. (2025). Stable isotope disequilibrium between soil bound water and soil bulk water – Implications for estimations of plant water sources. J. Hydrol. 650, 132544. doi: 10.1016/j.jhydrol.2024.132544 42574925

[B59] Yan B. Zhang Y. N. Xu J. L. (2023). Hydrogeochemistry and Water Analysis Laboratory Manual (Wuhan: China University of Geosciences Press).

[B60] Yang B. Wen X. Sun X. (2015). Seasonal variations in depth of water uptake for a subtropical coniferous plantation subjected to drought in an East Asian monsoon region. Agric. For. Meteorol. 201, 218–228. doi: 10.1016/j.agrformet.2014.11.020 42574925

[B61] Zeng F. J. Zhang W. J. Liu G. J. Zhang D. Y. Li X. Y. Zhang L. . (2020). Stable restoration pattern and sustainable management technology of main dominant vegetation in typical desert areas of China. Bull. Chin. Acad. Sci. 35, 709–716. doi: 10.16418/j.issn.1000-3045.20200420001

[B62] Zhang J. Feng N. Li J. J. Wang F. Wang K. L. Chen H. S. (2025). Water stable isotope evidence reveals the impact of soil thickness on mobile water recharge in karst hillslopes, Southwest China. Sci. China Earth Sci. 68, 1–12. doi: 10.1007/s11430-025-1648-7 30311153

[B63] Zhang H. X. Hultine K. R. Li X. R. Hou J. Q. Sun J. Y. McDowell N. G. (2026). Size-related decline in dryland shrubs is related to reductions in hydraulic efficiency and carbon assimilation and not nonstructural carbohydrate depletion. New Phytol. 249, 2273–2286. doi: 10.1111/nph.70615 41024511

[B64] Zhang J. Jia B. B. Zhang Y. H. Zhao D. M. Zeng X. D. Wang C. X. . (2014). Research progress on Calligonum L. in China. Gansu Sci. Technol. 30, 145–148.

[B65] Zhou H. Zhao W. Z. He Z. B. Tian L. H. Gu H. L. Fan M. Y. (2026). Hydrological niche segregation and species coexistence in arid zones. J. Desert Res. 46, 175–183.

